# Supplementary material for: E7-mediated repression of miR-203 promotes LASP1-dependent proliferation in HPV-positive cervical cancer
Source: Oncogene. 2024 May 24;43(28):2184–98. doi: 10.1038/s41388-024-03067-4 (PMC11226402; doi:10.1038/s41388-024-03067-4)
Supplement: Supplementary file 1 — Supplementary Figures [file 41388_2024_3067_MOESM1_ESM.docx]

**
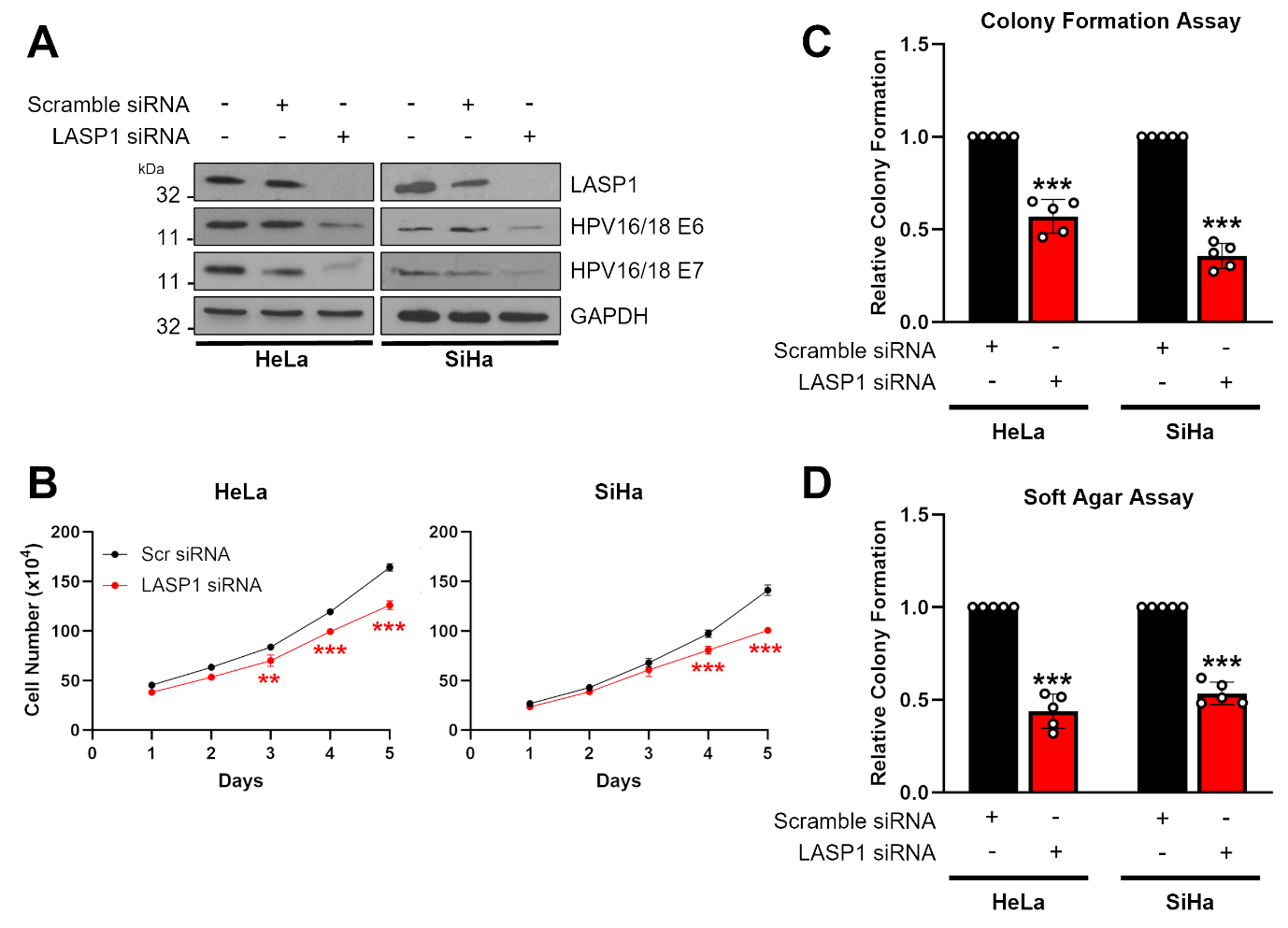
Supplementary Figures**

**Supp Figure 1. Transient LASP1 depletion inhibits proliferation in HPV+ cervical cancer cells. A)** Representative western blot of LASP1 protein expression in HeLa and SiHa cells after LASP1 depletion with a pool of four specific siRNAs. Lysates were probed for LASP1, HPV E6 and HPV E7. GAPDH was used as a loading control. **B)** Growth curve assay in HeLa and SiHa cells after LASP1 depletion with a pool of four specific siRNAs. **C)** Colony formation assay in HeLa and SiHa cells after LASP1 depletion with a pool of four specific siRNAs. **D)** Soft Agar assay in HeLa and SiHa cells after LASP1 depletion with a pool of four specific siRNAs. Error bars represent the mean +/- standard deviation of a minimum of three biological repeats unless otherwise stated. *ns –* not significant, **p* < 0.05, ***p* < 0.01, ****p* < 0.001 (Student’s *t*-test).

**
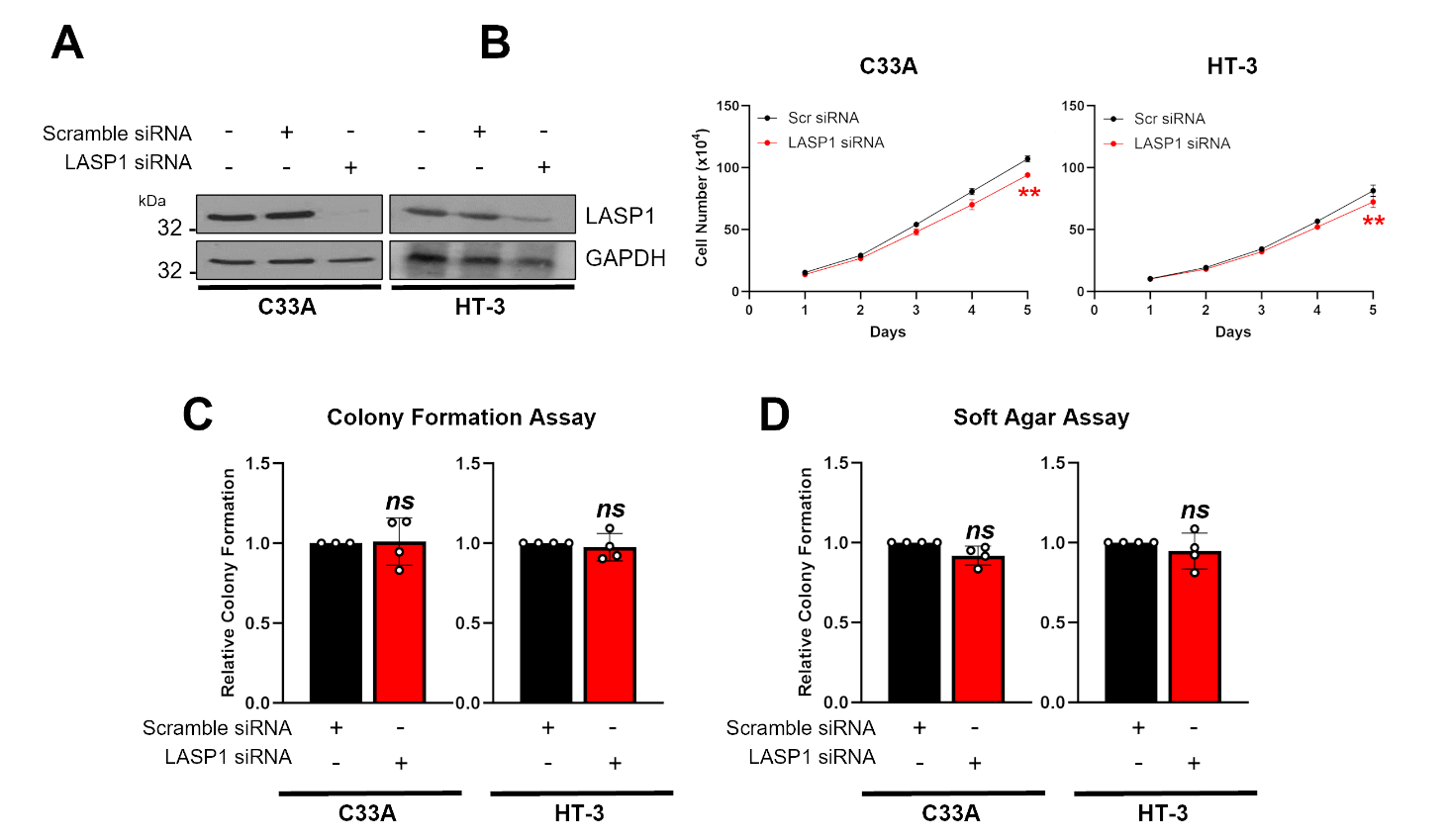
**

**Supp Figure 2. Transient LASP1 depletion has minimal impact on the proliferation of HPV- cervical cancer cells. A)** Representative western blot of LASP1 protein expression in C33A and HT-3 cells after LASP1 depletion with a pool of four specific siRNAs. Lysates were probed for LASP1 and GAPDH was used as a loading control. **B)** Growth curve assay in C33A and HT-3 cells after LASP1 depletion with a pool of four specific siRNAs. **C)** Colony formation assay in C33A and HT-3 cells after LASP1 depletion with a pool of four specific siRNAs. **D)** Soft Agar assay in C33A and HT-3 cells after LASP1 depletion with a pool of four specific siRNAs. Error bars represent the mean +/- standard deviation of a minimum of three biological repeats unless otherwise stated. *ns –* not significant, **p* < 0.05, ***p* < 0.01, ****p* < 0.001 (Student’s *t*-test).


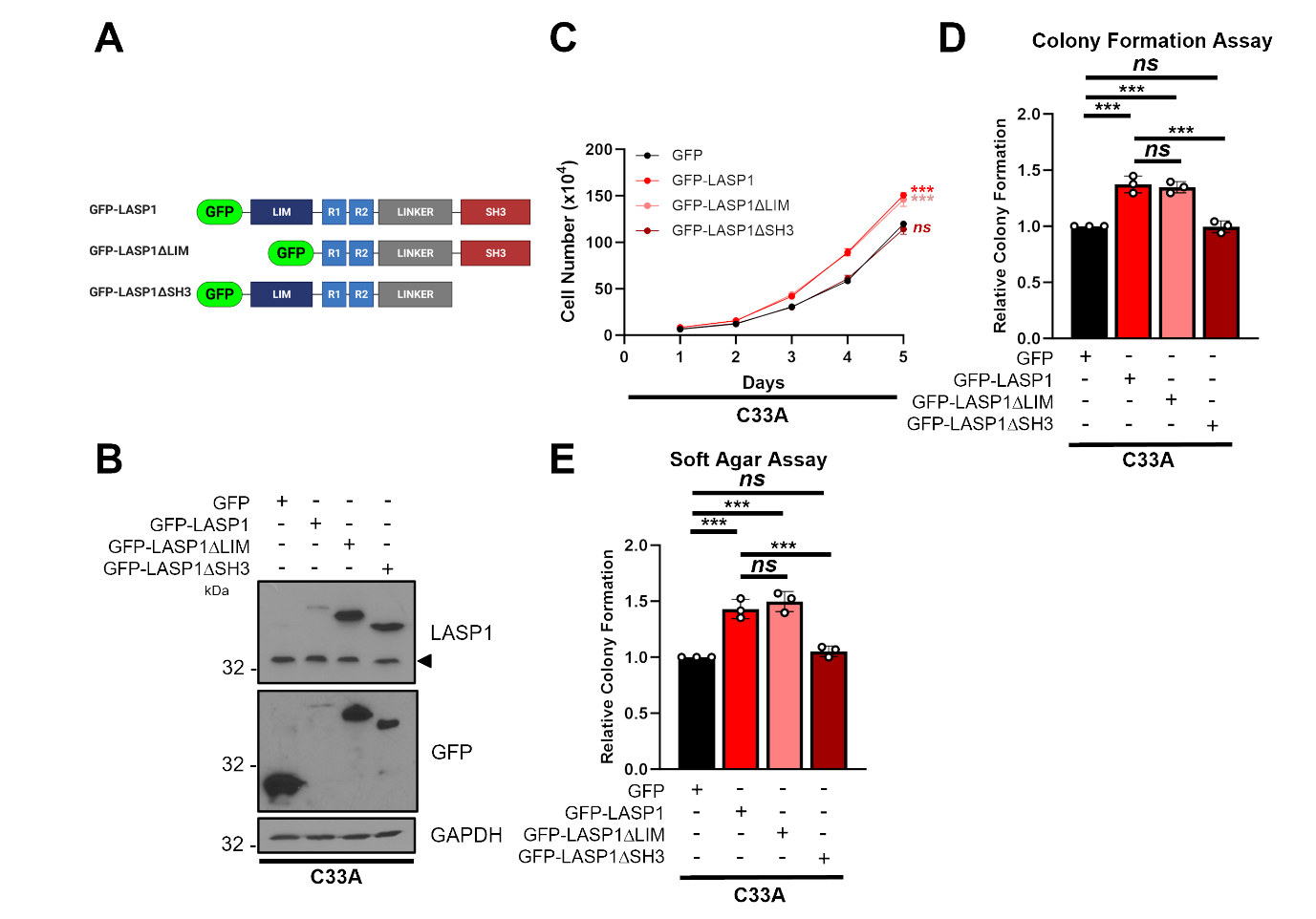


**Supp Figure 3. LASP1 promotes proliferation in HPV- cervical cancer cells in an SH3-domain dependent manner. A)** Schematic showing the LASP1 mutant constructs used in the study. **B)** Representative western blot of LASP1 mutants in C33A cells. GFP-LASP1, GFP-LASP1ΔLIM and GFP-LASP1ΔSH3 expression were confirmed using GFP and LASP1 antibodies. Arrow indicates endogenous LASP1 expression. GAPDH was used as a loading control. **C)** Growth curve assay in C33A cells expressing GFP-LASP1, GFP-LASP1ΔLIM and GFP-LASP1ΔSH3. **D)** Colony formation assay in C33A cells expressing GFP-LASP1, GFP-LASP1ΔLIM and GFP-LASP1ΔSH3. **E)** Soft Agar in C33A cells expressing GFP-LASP1, GFP-LASP1ΔLIM and GFP-LASP1ΔSH3. Error bars represent the mean +/- standard deviation of a minimum of three biological repeats unless otherwise stated. *ns –* not significant, **p* < 0.05, ***p* < 0.01, ****p* < 0.001 (Student’s *t*-test).

**
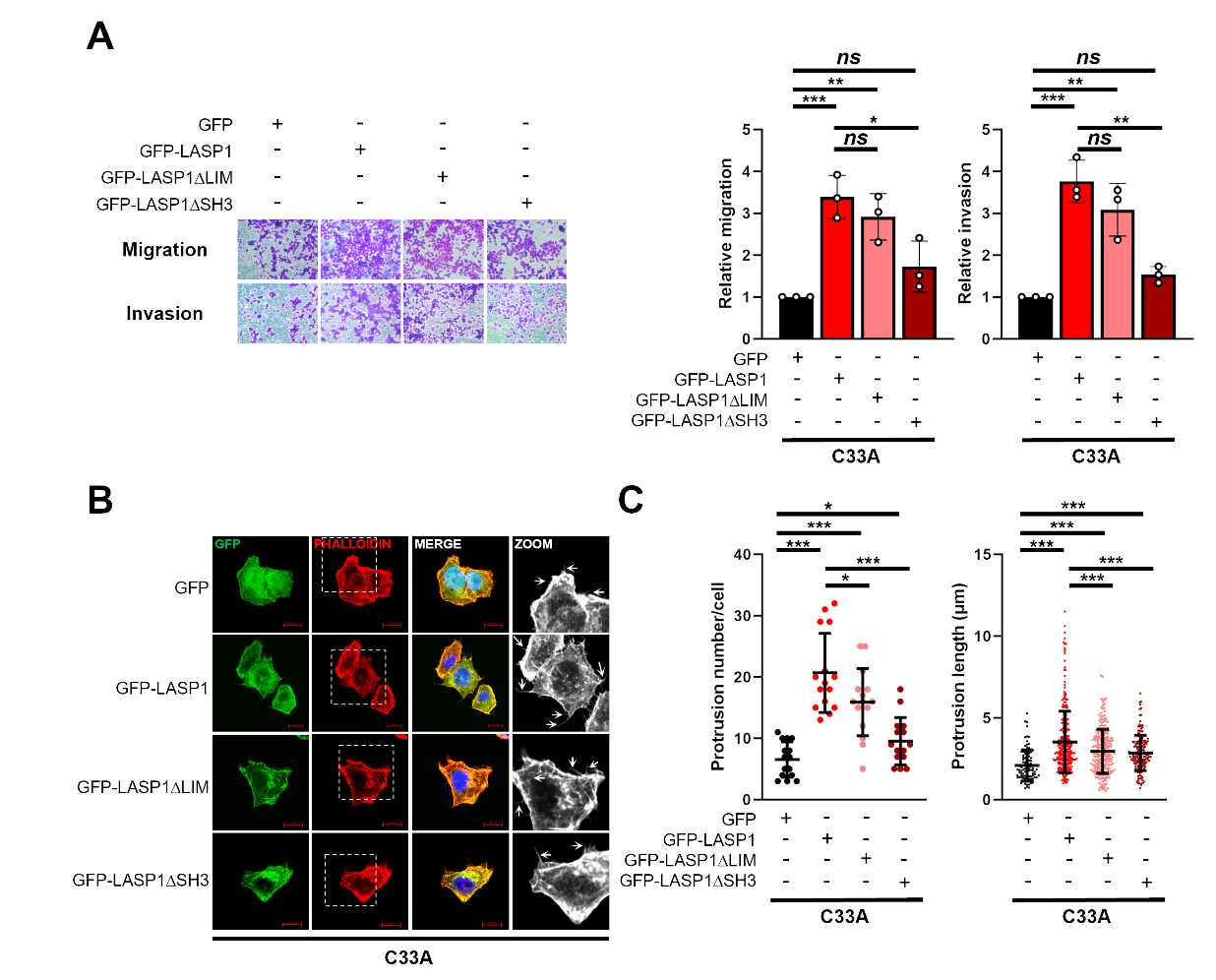
Supp Figure 4. LASP1 promotes the invasive phenotype of HPV- cervical cancer cells. A)** Transwell® migration and invasion assay in C33A cells expressing GFP, GFP-LASP1, GFP-LASP1ΔLIM or GFP-LASP1ΔSH3. Relative migration and invasion is demonstrated in the graph on the right. **B)** Representative immunofluorescence microscopy images of F-actin in C33A cells expressing GFP, GFP-LASP1, GFP-LASP1ΔLIM or GFP-LASP1ΔSH3 using Rhodamine-conjugated Phalloidin (red). DAPI (blue) was used as a nuclear counterstain. Scale bar 10 µm. Arrows point to F-actin protrusions. **C-D)** Number of F-actin protrusions **(C)** and length of F-actin protrusions **(D)** stained by Rhodamine-conjugated Phalloidin per cell in C33A cells expressing GFP, GFP-LASP1, GFP-LASP1ΔLIM or GFP-LASP1ΔSH3. Each protrusion counted in **C**) was measured using Image J (5 cells per replicate, performed in triplicate). Error bars represent the mean +/- standard deviation of a minimum of three biological repeats unless otherwise stated. *ns –* not significant, **p* < 0.05, ***p* < 0.01, ****p* < 0.001 (Student’s *t*-test).
